# Making genomic data FAIR through effective Data Portals

**DOI:** 10.1038/s41597-025-06142-x

**Published:** 2025-11-28

**Authors:** Matthew L. Speir, Wei Kheng Teh, Marc D. Perry, Rachel Schwartz, Parisa Nejad, Tim Harris, Clay Fischer, Mark Diekhans, Brian T. Lee, Benedict Paten, W. James Kent, Maximilian Haeussler

**Affiliations:** 1 Genomics Institute, University of California Santa Cruz, Santa Cruz, CA USA; 2 EMBL-EBI European Bioinformatics Institute, Wellcome Genome Campus, Hinxton, Cambridge, UK

**Keywords:** Databases, Databases, Genomics

## Abstract

Genomic data portals collect, annotate, and make data files available to researchers and, increasingly, AI algorithms. They are run by, among others, broad data archive repositories or consortium-specific Data Coordination Centers. Their design may seem a niche topic, but these portals realize the open data principles by making millions of data files findable, accessible, interoperable, and reusable (FAIR). Almost every researcher uses them, yet, we are unaware of published guidance on how web data portals should be funded, built, and run. We present lessons we have learned from creating genomics-focused data portals. We highlight the importance of funders in defining rules, human data wranglers as liaisons, a flexible and simple metadata schema, and a user-centered engineering process. We also present concrete suggestions on accessions, metrics, testing, controlled access, and licenses. Finally, we discuss the unsolved problems of interoperability, portal reuse, and long-term stability. We hope these guidelines can help funders and creators of new data portals develop a better understanding of the unique challenges they may face and possible solutions.

## Background & Summary

Centralized specimen collection and comparison, originally through museums, is one of the bases of biology as a science^1^. Data repositories (sometimes called digital archives) have continued that tradition and have been a part of the landscape of molecular biology research for nearly four decades. Two of the most prominent ones today, GenBank^2^ and European Nucleotide Archive (ENA)^3^, were founded in the early 1980s by the National Institutes of Health and European Molecular Biology Laboratory, respectively^1^. These were later joined by the Gene Expression Omnibus (GEO)^4^, BioStudies^5^, and many more that have often served as repositories for specific data types. In the last few decades, these large, general repositories have been supplemented by a growing number of data coordination centers, or DCCs, usually focused on organizing the data for a project, consortium or funding initiative. Examples include: the Encyclopedia of DNA Elements (ENCODE) Project Portal^6^, Gene Tissue Expression (GTEx) Portal^7^, Human Biomolecular Atlas Program (HuBMAP) Data Portal^8^, 4D Nucleome Data Portal^9^, National Alzheimer’s Coordinating Center^10–12^, (see Table S2). Some projects (e.g. NHGRI eMerge) store data in general data archives instead of developing their own DCC infrastructure and data portal. For controlled-access data, many National Institutes of Health (NIH) projects use the general data repository the Database of Genotypes and Phenotypes (dbGaP, https://www.ncbi.nlm.nih.gov/gap/)^13^ and Analysis Visualization and Informatics Lab-space (AnVIL, https://anvilproject.org/)^14^, which have data access committees that can review research projects and approve access requests^15^. Data that does not fit into any of these archives or DCCs can be uploaded to general scientific file archiving websites such as Zenodo or Figshare, which this article does not cover.

Both general data repositories and project-specific DCCs offer data files today through “data portals”. These accept submissions from research labs, validate and annotate them using a metadata schema, and then make them available to the research community through an interactive web portal and other means. Data portals strive to make research data available according to the FAIR Data Principles (Findability, Accessibility, Interoperability, and Reusability)^16^, which is crucial for scientific progress and research reproducibility^17–19^. Methods that leave labs in charge of data sharing (i.e. “data available upon request”) have fallen short of the ideal, aging data has been found to become quickly lost or corrupted^20–23^, and researchers increasingly accept sharing their data^24^. Funders have made data sharing a part of grant requirements as they believe that it allows their agency’s research dollars to go further in fueling scientific discoveries^17,25^. For example, the NIH requires data management and sharing plans in all research proposals and major research publishers, such as Nature Publishing Group, require authors to make their data available through repositories, DCCs, or general file sharing websites (see *Nature*’s “Data Availability” guidelines for authors). But despite data sharing requirements, problems with reproducibility and reusability are extremely common, as most researchers know from personal experience^26–28^. A well-designed data portal can help facilitate submissions, accurately represent the data and allow for easy downloads.

**Fig. 1:**
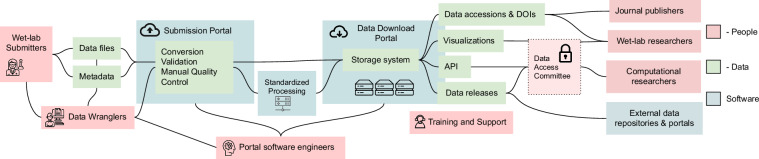
A typical data portal arrangement. Shows the stages from submission to release with the stakeholders and nomenclature for a data portal. Data files and metadata flow from the wet-lab submitters (left) into the data submission portal (center) with the help of data wranglers (bottom left). Once data is in the portal, it is converted, validated, and run through quality control (QC) tools (center), possibly through standard processing, often in a dedicated data center or the cloud. Data is then put into a long-term storage system (center), accessioned, and made available to researchers through a data download portal with data releases, visualizations, and more (right). The entire submission process is facilitated by the training and support team, who write the documentation and mediate between end users and software engineers.

This article focuses on providing practical recommendations for building a data portal that makes data FAIR and increases its broad scientific impact. These recommendations are based on the authors’ experiences from building more than ten data portals for DCCs over several decades (Table S1). Each section covers one facet of building and managing a data portal resource. At the outset, we offer an overview of the structure of a typical data portal (Fig. 1). The first two sections discuss the relationship between funding organizations, data submitters, and data wranglers. The next three sections focus on the process of getting data into the portal, annotating and accessioning that data. The following two sections cover how to make that data available and findable through a web portal. The final six sections explore various aspects of data management, analysis, and storage in the short-term and DCC management in the long-term.

Our key recommendations in this article are:

1. **Funders need to set and enforce rules for data sharing**. If there is no mandatory data release rule (e.g. “6 months after sequencing the data must be released” or “data must be released with publication”), then a lot of data may never be released or receive incorrect metadata.
2. **Data wranglers are integral to the submission process**. The data wranglers, human staff, act as the bridge between the data submitters and the portal’s database by helping facilitate submissions. As part of this, they help ensure consistency in metadata, annotations, and file formats between submitters. Without a team of humans who look at all the incoming data, it will be difficult to maintain consistency between submitters.
3. **The metadata schema needs to balance data reuse and submitter burden**. A metadata schema that is too simple may rule out some future uses, while a schema that is too complex can discourage submission and require extra assistance from data wranglers. To find a compromise, clearly defining potential reuses of the data upfront is important for creating a schema that accurately describes the data while enabling its future use. Uncommon metadata cases could be supported through free-form text fields, which may allow annotation gaps to be corrected at a later date by human curators or large language models.
4. **Ease of use needs to be considered in all aspects of the portal**. In addition to designing a balanced metadata schema, a data portal should aim for ease of use in all aspects of its design, from the submission process through its data portal. A portal engineering team can take advantage of user experience (UX) research to help define user profiles, identify clear use cases, and make the various interfaces accessible to users. For example, filters that require SQL knowledge, complex file identifiers, or downloads that recommend gridFTP will be difficult for many users, especially those without computational experience.
5. **There need to be multiple routes for accessing data**. In addition to offering a web portal with interactive downloads, a data portal should offer programmatic access to its data. This can be through an API, FTP, or other means. These access routes need to address the needs of a data portal’s varied user groups. For example, an interactive website makes data accessible to those without computational experience and an API, FTP interface, or other command-line accessible endpoints make data accessible to those performing computational analysis of the data.
6. **The engineering team needs to remain responsive to changes**. Assays and assay technologies will evolve and shift over the life of a project. A data portal must factor this potential for change into its release process and be responsive to these changes, both in its metadata schema, file format and software design. Customized free form fields should be allowed everywhere, rather than be designed to be an exception.
7. **Controlled-access data and consent forms need to be factored in from the very beginning**. While not all data portals will host such data, for those that do it will affect all aspects of the software design, including submission, data storage solutions, and the data portal. If it is not considered from the beginning, it may be difficult or impossible to retrofit a data portal to make controlled-access data available.

## Stakeholders and structure of data portals

Data portals host an incredibly wide variety of results derived from a diverse set of molecular assays and imaging techniques performed on the transcriptome^4^, proteome^29^, epigenome^6^, or metabolome^30,31^ of single cells up to whole tissues. Some portals, such as UK Biobank^32^ or All of Us^33^, are creating databases of longitudinal data that combine diverse collections of experimental data with patient healthcare information over time. Despite the distinct focuses of all these different portals, we believe that there are a few guiding principles in designing and supporting a data portal that can be followed to make the data FAIR.

### Funders

#### Goals

Funding organizations play an important role in the success of a DCC or data repository. Funders should have clear goals for a DCC or repository data portal before the project starts. Data portals are often the main, public-facing product of a consortium beyond its publications, and so consortium funders should have a vision for this product. For example, the National Institute of Mental Health (NIMH) may require its portals to use the institute’s NIMH Data Archive (NDA) or the BRAIN Initiative’s assay-specific archives as well as adhere to specific metadata standards for future integration. Smaller funders, such as Alex’s Lemonade Stand (https://scpca.alexslemonade.org/), may participate more in the day-to-day management of the portal or run them entirely in-house. A funding organization should have staff or advisors with experience in both IT and biology to help with creating clear and relevant goals. Many data portals start with unclear use cases. Funders should define a concrete list of tasks that researchers in the domain expect the portal to solve and should be sure that there are enough users, by running surveys or conducting at least minimal demand assessment. The funder is also responsible for selecting the team that will manage the data portal, ideally one with at least some staff with experience in the domain.

#### Standards

A funder sets the parameters for the relationship between the DCC and data submitters. The CSER DCC called this relationship between its various DCC stakeholders, including data wranglers and data submitters, its “team morale” and thus sought to foster a sense of mutual respect between these stakeholders^34^. The funder determines standards for data sharing in the consortium and what action should be taken if these standards are not met by submitters. The standards need to be defined clearly and communicated to all labs. They should be as specific as possible, e.g. “all raw genome reads must be submitted within six months of the completion of sequencing.” A vague rule such as “three months after QC” can be interpreted by labs as “one day after publication”. Without clear submission rules, even the best data portal will have little data to show for years and will be powerless to enforce the rules without direct funder intervention. Finally, if patient consent is involved, the funder must determine and communicate consent guidelines before the project starts and then enforce these throughout the project, because poorly designed patient consent forms can prevent any data sharing forever.

#### Carrots and sticks

As part of setting these standards, the funders should consider the “carrot and stick” approach. The “carrots” are positive incentives for the labs to share their data and submit high-quality metadata. For example, the DCC can keep the data structured and secure for future researchers, make it easily accessible to journals and reviewers, or provide data visualizations and data summaries. A portal can also offer to submit certain data to general repositories such as NCBI SRA or ENA on behalf of the labs. “Sticks,” on the other hand, are the funder’s clear, defined data sharing standards discussed above and the possible sanctions that may be imposed for not following these standards^23,35^. While most groups will voluntarily submit their data to the portal, in our experience, a funder will always need to intervene at some point to convince some research groups to submit data on time and provide sufficient and correct metadata. Also, in our experience, whenever there is any project-wide deadline for labs to submit their data to the data portal, expect the majority of the data to come in a few days before such a deadline, so deadlines should ideally be spread out over the submitting labs.

#### Enforcement

The funder is often the one to enforce rules regarding data sharing. For example, the CIRM Center for Excellence in Stem Cell Genomics project required labs to use specific sequencing centers who would submit data to the DCC on their behalf. To obtain the data from the data portal, the lab would have to submit some basic metadata for their sequencing files and only then get access to the results. Despite sometimes incomplete metadata, the data wrangling team was at least aware of any genomic data generated during the project. However, a lot of data was never released publicly on the data portal. ENCODE went one step further: it required that all data must be released publicly after an embargo period of six months (which was eventually removed entirely). A “dashboard” of the amount of promised data per quarter per lab and actually submitted data has been implemented by the BICAN and SSPsyGene projects to check compliance. In rare cases, such as the Human Cell Atlas DCP, all submissions are voluntary. In such a case, there simply is no stick at all. The data portal team will need to plan to have more carrots available, a larger budget for marketing and outreach, and rely on gentle prodding of labs to start, fix, and complete their submissions.

#### Budget

Funders and the groups tasked with managing a data portal should be aware of the costs. Based on data available in the NIH RePORTER (https://reporter.nih.gov/), a database of NIH-funded projects including their costs and outcomes, the NIH seems to plan for around 10% of the total budget of most consortia to be used for the DCC. For example, the ENCODE project, active from 2003 to 2021, set aside around three to four million US dollars for building its DCC, not including the costs for its uniform analysis pipelines, out of a total budget of roughly thirty million US dollars. The numbers are similar for now completed “The Cancer Genome Atlas” (TCGA), 3 of 33 million US dollars, and the ongoing “Impact of Genomic Variation on Function” (IGVF), 4 of 37 million US dollars over five years (https://reporter.nih.gov/project-details/10849674). These are just a few examples out of many dozens others that illustrate that data portals consume a non-negligible portion of “big biology” budgets, often the equivalent of one or two data generation centers.

### The role of data wranglers

Data wranglers serve as a bridge between the submitters and the data portal. They guide research labs through the submission process, from metadata collection to data upload and file annotation. The data wranglers can also be understood as the “gatekeepers” of a portal’s data to help ensure its FAIR-ness. A data portal without human wranglers, e.g. the Broad Single Cell Portal, will have metadata of highly variable quality. A recent study analyzed metadata for COVID-19 samples submitted to GenBank, ENA, and BioSamples and found considerable variations in how the disease was labeled, e.g. *COVID-19* vs *COVID19* vs *novel coronavirus pneumonia* and this slowed down data usage^36^. Wranglers ensure data files are correctly annotated with sample or patient attributes or assay details to ensure their future reuse. One of the primary values of a data portal comes from its ability to make data comparable across submitters by organizing it and describing it with an adequate metadata standard. If the metadata is not comparable, re-analysis across datasets becomes increasingly difficult. The efforts of one curation team to harmonize data as it is submitted increases its FAIR-ness by making it so that every data consumer does not need to spend time doing this harmonization. Data wranglers are important beyond assisting with the submission process. They can use researcher feedback to improve that process by adjusting documentation, examples, and standards by extending controlled vocabularies or ontologies and guide the software engineers. To ensure consistency when capturing metadata across projects, data wranglers depend heavily on their data portal metadata standards, also known as the “metadata schema”.

### An appropriate metadata schema

Metadata is information about the files themselves, such as sex, sequencing protocols or sample sources, that play an important role across the life of a dataset. Metadata can highlight new patterns during analysis, it can assist in quickly finding relevant data in a database, and it can be essential for reproducibility. Clear metadata is essential for finding relevant data in the millions of files in a public database. Finally, metadata is crucial for reproducibility, such as for cell type annotations in single-cell sequencing studies^26^. If important information is not added when the metadata is collected, it is a time-consuming manual effort to add these details at a later date using publicly available sources, if possible at all^37,38^. Reannotation efforts can require the involvement of the original submitters, which may be impossible when the staff that originally produced the data (e.g. postdocs or graduate students) have moved on.

#### Minimal and customizable

A portal should employ a robust suite of metadata systems to help with harmonizing technical terms across researchers and agencies – ensuring that everyone is speaking the same language. This harmonization should take advantage of existing ontologies and clearly-defined controlled vocabularies. For example, the National Cancer Institute’s Genomic Data Commons (GDC)^39^ and ENCODE^40,41^ provide a set of structured schemas to describe both the data and post-submission analysis. Existing “minimal information” (MI…) standards may be used as a starting point; they exist for microarrays (MIAME^42^), genomic experiments^43^, images (MITI^44^), and general biological investigation (MIBBI^45^). Frameworks such as Phenopackets^46^ or the HIPAA-compliant REDCap^47^ provide a starting point for groups that need to gather and collate patient data. However, if there are too many fields in the metadata schema, then submitters will have a hard time using them. The NIMH Data Archive (NDA) for example has more than 2,000 data structures and 300,000 possible fields - it is unlikely that anyone will be able to look over them to find the best ones for a submission (https://nda.nih.gov/general-query.html). Most portals use a three-tiered approach to metadata: (1) required core fields, (2) optional fields, and (3) user-defined fields. This structure allows some flexibility in adding new fields to the schema at the request of submitters. A core requirement of the software is flexibility, and user-defined fields are one way to make sure that submitters can enter any data and that the software and metadata schema can be improved later to harmonize these, so every schema should have customizable fields.

#### Small

For metadata standards, bigger is not always better. While it is important to have a robust metadata standard, it needs to be weighed against the submission burden it imposes on submitters. Harmonization rigidity will depend on how the data is intended to be used in the future, the available funding and curation staff count, the amount of time submitters are willing to dedicate, the amount of data expected, how long data can stay in the curation queue, and the complexity of the data covered by the  data portal. A metadata schema for a project with few submitters but a diverse array of data types (e.g. ENCODE, CSER) will likely be harder to design than a project with dozens of submitters but very similar assays (e.g. TCGA, GTEx). The hardest parts to standardize are those where research is ongoing or assays are being developed, e.g. for brain cell types or new types of sequencing or new types of imaging assays. It may be easier to use customized, free-form fields for a while and harmonize datasets post-hoc instead of trying to define standards that cannot be defined yet.

#### Tooling

A complex metadata schema can place a significant burden on submitters. To assist data generators with their submissions, a portal should provide a set of easy-to-use tools that submitters can use to fill out and check the metadata. For most datasets in both the Human Cell Atlas (HCA) Data Coordination Platform (DCP) and the 4D Nucleome, it is the data wranglers who fill out the metadata spreadsheet. However, the research labs are able to at least provide a draft version of the metadata for the data wranglers to work from. The submission burden in these cases could be lessened by providing the data generators with better tools for supplying metadata, similar to those provided by GenBank (https://submit.ncbi.nlm.nih.gov/about/genbank/) or ENCODE (https://www.encodeproject.org/help/submission/). These submission tools can also help automate the validation process for both data files and metadata, saving both the wranglers and the submitters time.

### Data validation

A data portal will need to include systems to validate submitted data files before making them available to users. Submitted files should be validated at least to ensure that they are uncorrupted. It is even better if this file type validation is combined with metadata and used to alert wranglers to issues with a submission (e.g. sequencing data labeled as ‘human’ should not match the mouse genome). Studies of public transcriptomics archives have widespread issues for even metadata fields as simple as “sex” for which one study found errors, so discrepancies between the metadata and genome-determined sex, in around 46% of projects they surveyed^35,48^. Several of the DCCs examined for this paper as well as major data repositories like GenBank employ automated pipelines to validate files and alert submitters or data wranglers to issues. The HuBMAP Consortium DCC employs a suite of tools for validating submissions including over 170 “automated and manual QA/QC checks as part of the data submission & publication process” (see “HuBMAP Data Validation” at https://docs.hubmapconsortium.org/technical.html). And modern contamination screening tools can now quickly detect the organisms in sequencing files^49^. Regardless of the methods employed, robust validation can help find and flag data file and metadata errors early as data needs to be correct and uncorrupted to be FAIR. However, QC procedures beyond the basic file format checks can be time consuming to program and update, and given constrained IT resources, in many cases a fast and simple data portal may be a higher priority than extensive QC checks.

### Data accessioning

A data portal should assign unique identifiers that can be referenced in manuscripts to help ensure reproducibility and encourage reuse in accordance with the FAIR principles. In biology, these unique identifiers are typically called “accession numbers,” a term known from libraries. While some only assign accessions at the project level, many portals, including both ENCODE and 4D Nucleome, opt to accession each project, each file, library, and more. Accessions should be simple and unique: a short DCC-specific prefix followed by a set of letters and/or numbers. A unique, specific prefix, such as “4DN” for 4D Nucleome or “HBM” for HuBMAP, will make it easier to find mentions of the data with web search engines later. The number of digits should be long enough to be unique, but not overwhelming to the user, especially when there are many consecutive 0 s. For example, RefSeq (e.g. NM_002500) are easier to type than Ensembl accessions (e.g. ENSG00000162992). Alternatively, an alphanumeric identifier can be used and even be made more memorable by making them pronounceable. Less ideal are those that are just short strings, such as those from Entrez (e.g. 4760) or Protein Data Bank (e.g. 2QL2). The structure of these accessions make it difficult to recognize them as accessions and will make it harder to track down citations later with search engines. With a prefix, the accessions, e.g. “PDB:2Ql2”, are easier to recognize and find with software. Data portals should have short URLs for accessions for easy citation as short URLs of <20 characters are less likely to get mangled by line breaks in a journal PDF. A data portal can simplify the citation process further by offering DOIs through a service such as DataCite^50^. This allows authors to put links to datasets or files into their publication using the bibliographic reference managers of their choice. Hundreds of university libraries offer DataCite DOIs for free to interested data portals, see https://datacite.org/members/.

### Data access and licenses

#### File formats

A data portal must think about how to make its data accessible according to FAIR Principles. An easy-to-use API (usually RESTful^51^) and a user-friendly web interface (see next section) are common today. Data files should be made available through standardized, broadly-used file formats to reduce the burden on those consuming the data. When possible, these files and interfaces should follow the guidelines put forth by the Global Alliance for Genomics and Health (GA4GH), which proposes specific formats, APIs, and architecture to promote interoperability^52^. For example, the 4D Nucleome Data Portal provides both a REST API and an intuitive website that provides data in bigWig, bigBed, FASTQ, and many other standard formats. Beyond APIs, metadata should also be provided as tables (e.g. in TSV or SQL formats), which simplifies the task for labs that download data in bulk and meta-archives that index several portals. These can also be transferred to other portals when the end of funding is reached (see “Unsolved challenges”).

#### Open licenses

Whenever possible, a data portal should make data available under an open license that allows redistribution without enforcing special acknowledgments or other constraints. Restrictive licenses make it difficult for the data to be integrated into other databases or made available on data portals for visualization and analysis. For example, much of the data in infectious disease databases is stored in a single database with restrictive licenses, GISAID for flu and COVID-19 sequences and cannot be redistributed, copied, or backed up elsewhere^53^. Its license limits access and redistribution of its data to only those with a GISAID account (https://gisaid.org/terms-of-use/) or explicit approval by the founder of the database. This means that no one can combine the data with public data in GenBank or provide analysis tools for the biggest collection of Sars-COV-2 sequences which is GISAID. In contrast, most genomics data is made available under extremely permissive licenses. For example, the HCA DCP uses a “CC BY 4” license, meaning that the data can be shared or adapted for any purpose (https://data.humancellatlas.org/about/data-use-agreement). The ENCODE project has a similarly broad data use policy that states that “all data produced will be available for unrestricted use immediately upon release to public databases” (https://www.encodeproject.org/about/data-use-policy/). This means that anyone can extend or improve these datasets and publish new tools or visualizations for them.

### Designing a data portal website

The data portal website is one of its most crucial data systems in making the data FAIR. Importantly, it enables the discovery and download of relevant data and should do this using a variety of methods to account for the different ways people imagine the data. One of the most straightforward is the use of faceted browsing, now a staple of e-commerce sites. Faceted browsing allows researchers to drill down to the most relevant data using a combination of well-known terms and free-text search. This can be supplemented by providing themed pages around diseases, organisms, labs, or other key delimiters to facilitate finding relevant data. The ENCODE project uses an “experiment matrix” to show all assays and file types on a single page **(**Figure S1). Almost all portals that we know, ENCODE, HCA, GDC, NEMO, and 4D Nucleome projects, to name just a few, allow both free text search and faceted filtering to allow users to drill down to the data of interest (Figure S2). Some portals organize their files into “datasets,” typically analyzed and interpreted together. If this is the case, datasets ideally include well-written description pages with interactive figures to make them accessible and understandable (Figure S3, S4). Some portals go even further, such as ENCODE and 4D Nucleome (Figure S5), and include detailed file pages that display QC metrics, file provenance, and pipeline diagrams. Data should ideally be presented to researchers in a way that allows them to understand how it was generated and determine its quality before downloading it.

#### User interface design

It is challenging to design a website and supporting software tools that meet the diverse and sometimes divergent needs of both wet-lab biologists and computational analysts. Addressing both of these groups is important and may require distinct feature sets. For example, wet-lab biologists may want to be able to download data through an interactive interface but computational analysts would likely prefer to retrieve the data using Unix command line tools or an API. It can be difficult to discern the unique needs of the various user groups. Professional user experience (UX) designers, either as part of the software team or hired via contracts, can be employed early to help conduct surveys and other research to help understand user needs. For example, UX research from Human Cell Atlas Data Coordination Platform found that wet-lab scientists often do not want to solely depend on computational biologists for basic analysis and appreciate when a web interface provides the option to perform some basic analyses and visualizations (e.g. differential expression). This requires software engineers who understand the particular data types and analyses, a topic where sometimes even the scientists may not agree on the best parameters.

### Standardized versus submitted analysis

Most data portals provide certain analysis results alongside the raw data, either the output from standardized pipelines or results from submitters themselves. For example, a DNA sequencing project will usually provide genome variants while a big RNA sequencing project will usually provide gene expression tables. These analysis results should be provided in standard, commonly-used formats whenever possible (e.g. VCF for variants), making the data easier to reuse and interoperable with existing toolsets. Even though the ENCODE Project includes data from a wide variety of genomics assays, they were able to create a set of ‘Uniform Processing Pipelines’ to analyze some of their major assays, including RNA-seq, ChIP-seq, ATAC-seq, DNA methylation, and several others. The NCI GDC applied uniform analysis pipelines to specific data types^54^. In other cases, the sheer range of experimental assays or organizational structure may prevent the use of standardized pipelines. For example, both GEO and the BICCN (https://biccn.org/), which cover an even wider range of assays, have chosen to host only submitter-supplied analysis files and not re-run pipelines. The Alex Lemonade Stand collected raw data first into a portal and then embarked on a multi-year project to analyze it collectively with various research labs and make the results available in the same portal. Most general data repositories do not run any analysis, e.g. SRA or ENA. However, GEO allows submitters to upload their analysis as “supplemental files” and, as a result, GEO now contains millions of files with thousands of different file types. Even if not storing analyses, a portal can provide ways for users to run cloud-based workflows on data (see next section) for those users who need to re-run the analysis with different parameters (e.g. different gene sets), or entirely different analysis algorithms not foreseen when the portal was set up.

### Cloud-based analysis and storage

The cloud offers both opportunities and particular challenges for data portals. Using cloud-based computing, a research group can access hardware beyond what their institution has available, which allows them to quickly scale up analysis capabilities beyond even the biggest academic clusters and almost infinite storage is available today. However, this flexibility comes at a cost. While CPU hours are relatively cheap, cloud-pricing models include fees for storage and outgoing data transfer that can fluctuate and usually exceed costs for locally managed storage. For example, the 4D Nucleome group reported in 2019 that they struggled with the long-term costs of maintaining their data in the cloud. Solutions to help optimize cloud workflows to reduce costs exist, but many fail to compare cloud computing costs against locally managed solutions^55–58^.

#### Download costs and hybrid approaches

If a group is considering using the cloud for analysis, the full cost of this approach should be calculated precisely, and include all downloads and compared against an on-site solution in an academic data center. Note that downloads from universities into the cloud are almost free, but the opposite is not true, there is a very high one-way barrier around all clouds that can quickly eat up grant budgets. The internet was built by and for universities and they connect to the main internet backbones for fixed monthly rates, unlike commercial clouds that charge per gigabyte. Concretely, downloading from a server located at a university is free for both academic compute cluster users and cloud users while data stored in the cloud is somewhat faster to access from servers rented from the same cloud vendor but costs around $60,000 to $90,000 per petabyte to download one single time to a university. Therefore, some portals allow both use cases, e.g. NEMO stores a copy of all public data in the cloud. If cloud support is a requirement but on-site hosting preferred, we note that most cloud providers offer free storage for some academic groups, such as Amazon’s Open Data Program (https://aws.amazon.com/opendata/). Their application forms are short and approval rates high. DANDI (https://www.dandiarchive.org/), for example, uses this program to keep one copy of the data on the cloud. If controlled-access is an issue, there are now cloud-based tools (e.g. Terra, https://terra.bio/) that fulfill US requirements for processing controlled-access data.

### Complexity of controlled-access data

A data portal intended to house identifiable data from human donors must consider how access to that data will be managed and safeguarded from the outset of its design. There are two aspects to safeguarding protected health information: patient consent and the technical systems that control access to that data. On the one hand, a data portal will have to manage the storage and access of that protected health information in a way that complies with local regulations. On the other hand, it will need to work with its funder and research groups to ensure that submitters obtain proper consent to allow redistribution of the data. If proper consent is not obtained, it can be impossible to ever redistribute any data even if all technical systems comply with local regulations. Given the complexity of securing these systems and setting up “data access committees” (see below), it may be easier for a project to deposit protected data with archives that are already equipped to handle such security requirements and have committees in place, such as the Database of Genotypes and Phenotypes (dbGaP) and the European Genome-phenome Archive (EGA).

#### Open summaries

It should be a goal from the beginning when writing the consent forms to allow release of at least some part of the dataset or metadata publicly, while keeping private health information protected. As much as possible, public summary-level data, such as fully open subsets or variants above a specific cutoff, can often be made available so that a project can advertise the data to researchers who may be reluctant to go through the data access process. For example, the UK Biobank releases summary statistics of genotype/trait associations and a global linkage disequilibrium matrix but keeps all individual genotypes under controlled-access.

#### Storage requirements

If existing archives cannot be used, the portal will have to refer to the various levels of regulation that govern the security of systems handling protected health information. A data portal must follow legislation that applies to hospital data systems^59,60^. In the United States and the European Union, DNA sequencing reads are considered personally identifiable and can only be handled on IT systems that fulfill strict requirements^59–61^. Among these requirements are specific encryption methods, mandatory security plans and their regular review, detailed logging, off-site backups, and restriction of physical server access. For example, for United States federal contracts, these security rules are defined in the 45 CFR Part 160 and can be fulfilled by following FISMA certifications and the special FedRAMP standards for cloud services. While these specific regulations would apply to groups in the US, DCCs in other regions would need to confer with their funders and local authorities about specific regulations that apply to them. For example, protected data from Germany must be stored within the country, such as with the German Human Genome-Phenome Archive (GHGA, https://www.ghga.de/). The same applies to many other countries, e.g. China and India.

#### Data access committees

As part of designing these technical systems, a data portal must also outline a clear data-access policy. It will have to answer difficult questions about who will be responsible for running a Data Access Committee (DAC). These check incoming requests from researchers for download against the data use restrictions and verify that the researchers exist and have the means to conduct the project. The two major data repositories for restricted access data, dbGaP and EGA, take a different approach. EGA requires data to have a DAC associated with it, although a single DAC can control multiple submissions. Access to data in dbGaP is controlled by a central NIH DAC, with submitters outlining the acceptable types of use. Ontologies such as the Data Use Ontology (DUO) allow submitters to use a standardized set of terms to describe data access rules, which may make decisions by a DAC easier, regardless of which option it chooses^62^.

### Early stress testing

Early in a project, submissions will be slow as data generators ramp up their work. Many DCCs the authors have built received only a few submissions during the first couple of years of the project. Submissions will peak sometime in the middle of the projects as experiments start to bear results. Most tend to come in a few days before any deadline. They will slowly taper off as the project or funding initiative comes to a close, but in our experience often continues to come in long after the project’s scheduled end and when DCC staff may be limited or has moved on. The slow rate of submissions early on may tempt a portal team to create a detailed metadata schema and many QC checks. However, these may not be sustainable during the submission peak and, likewise, the technical infrastructure may not be able to scale. This can be prevented by early stress tests using artificial submissions, to make sure that the IT infrastructure and the data wranglers can handle the load.

#### Flexibility

A data portal needs to remain agile and be willing to adapt its schemas and processes to new assays, plan for sudden spikes in submissions, and navigate the changing demands of the data generators. This flexibility is helped by adopting an iterative development approach with frequent, small releases and the facilitation of direct contact between data-generating labs, data wranglers, user interface experts, and software engineers, all supported by user outreach and training^34^. There are various project management schemes a project might use during development, including Agile or Lean among several others. We have worked across teams that employ various approaches and have not found there to be a significant advantage between them. A team will find out over time which project management style works for them.

### User support, outreach, and training

A data portal group should support its users in multiple ways, including a help desk, clear online documentation, tutorials, outreach, and training. All of these avenues of support complement one another. The support they can provide may also go beyond just accessing hosted data and help guide users to better understand metadata best practices, data protection, consent, and more. Through search, a homepage and regular email updates, users can first be guided towards step-by-step documentation, supplemented by more technical documentation. If questions still arise, users can reach out to the help desk for clarification (Figure S6). Help desks are there to provide support not just to researchers looking to start their submissions, but also to those looking to reuse the data. In addition to providing documentation and a help desk, portal teams often engage in training and outreach where they go to institutions or conferences and lead workshops on submitting data, accessing data, or utilizing other resources. The ENCODE project has held several workshops on utilizing the data in their DCC as well as some relevant specifically to consortium members. HuBMAP has created a Massive Open Online Course (MOOC) to communicate how data from across diverse labs is integrated and how one might use HuBMAP data in their research. The NCBI databases organize regular “Hackathons” and Ensembl has been running a “geek for a week” program for more than a decade, where data users come to the EBI offices. All of these options can feed into one another, with help desk questions highlighting gaps in documentation or features to highlight during training sessions. To ensure long-term support and success, a portal should try to secure funding for minimal staffing for at least a few years after the project has ended. The ENCODE Portal, for example, has been able to obtain funding for at least one employee for several years after the project’s end. Otherwise, long-term funding of a project-specific portal is very difficult to address (see “Unsolved challenges”).

### Usage metrics

Tracking the usage metrics can be used to measure the broader impact of the  data portal and the data it hosts. It can also be used to help justify the continued funding of a DCC web portal alongside other data access modalities, optimize usage, and focus curation efforts^15,63^. A DCC should combine a variety of metrics, at least publication citations, file downloads, and portal users. If a data portal makes use of short URLs, DOIs, and accessions (see “Data accessioning” above), these can easily be tracked using publication search engines, such as Web of Science, Scopus or Google Scholar. As long as the name of the portal website is unique enough (e.g. HuBMAP), mentions that are not citations can also be reported. Sometimes authors will mention a resource, but not cite its publication. For example, the various RefSeq papers are cited around 20,000 times, but “RefSeq” is mentioned in almost 100,000 papers, a five-fold difference. A very generic name, such as “ENCODE” makes publication tracking harder, as the word is used in English. Ironically, a name that is hard to pronounce, such as IGVF or SSPsyGene, make publication and web mentions easier. Downloads and users are usually tracked using invisible additions to the portal website such as Google Analytics or one of many similar tools, e.g. Baidu Analytics in China, where Google is blocked, or open source alternatives. Web analytics tools can also provide other insights, including the rough geographic location of users, the path they take through a website’s pages, the interface elements users interact with, and a website’s most viewed pages. All of these metrics can help with quantifying data reuse for reporting to funding organizations to demonstrate the utility of the portal. And, finally, these metrics can also help highlight the small number of frequently used datasets to help focus possible re-curation efforts^63^ or reduce storage speed and cost for rarely accessed datasets.

## Unsolved challenges: Interoperability, reuse and long-term funding

### Interoperability

Biologists face an increasing number of data portals that sometimes overlap in function, focus, and tooling. For example, a group performing human and mouse single-cell RNA-seq for the Human Cell Atlas with partial NIH funding has to submit mouse reads to NCBI SRA, human reads to dbGaP, expression data to GEO to fulfill NIH mandates, and single-cell data to the HCA DCP (sequences) and CELLxGENE (visualization) to fulfill HCA/CZI funding requirements. Each of these require different logins, signups, staff interactions, data transfer tools, and metadata schemas. Collaboration between data repositories and portals could build connections to lower the burden of submission on researchers, and store data only once or at least link between the data portals. The HCA DCP is making some headway on this front as they will submit suitable projects to visualization portals such as EBI Single Cell Expression Atlas or CELLxGENE. But often the burden is placed on research labs, which have to spend more and more time learning different tools and appropriate metadata schemas. We know of very few software packages and only for very specific use cases, that prepare an entire submission in an automated way, e.g. for SARS-Cov-2 sequencing data submitted to the ENA^64^.

### Reusable software

Reusable software tools could mitigate the duplication of work. On the administrative side, every portal has different levels of data protection, consenting, and access types. On the IT side, almost all genomic portals to our knowledge are bespoke solutions, meaning that research funders are expending effort on designing and maintaining similar systems. We are unaware of a single reusable software for preparing submissions to a data portal, for annotating metadata interactively, or validating genomic files before submission. In the field of patient forms and surveys, the REDCap system^65^ has shown that having a single system can lead to wide adoption and set standards across many clinical trials and internationally very quickly, but no similar effort exists for genomic data portals, to our knowledge. Having components that data portals could use in their development workflow would lower the cost for all of them and, in addition, would allow data to be more easily transferred. The DataBiosphere is an ongoing effort to create a set of reusable modules for cloud-based biological databases and is working to implement it in a handful of large projects such as TOPMed and All of Us^66^, but looks more of a concept than actual software. The Alliance of Genomics Resources is working on creating reusable software components for a future comprehensive model organism database^67^. The Overture Stack (https://github.com/overture-stack), maintained by the Ontario Institute for Cancer Research, appears to be another step towards reusable data portal components. But at the time of writing, research funders are standing up a new DCC for every consortium, rather than creating a single DCC software system or reusing existing DCC groups or extending existing general data repositories for a new consortium. The cost of the existing DCC and repository landscape and possibilities for economies of scale through reusability and centralization would merit further examination.

### Long-term sustainability and funding

As a result, more and more new data portals are coming online, partially driven by a push towards cloud-based solutions from NIH, more big-data assays in biology, e.g. the BRAIN project portals NEMO, BIL, and DANDI, for new types of sequencing, imaging and electrophysiology data, respectively, and restrictions on data transfer to foreign countries (e.g. Germany’s GHGA or the Chinese Bio Data Bank). Some of the project-specific DCCs among them will eventually reach the end of their grants and will need to shut down, unless bridge funding can be found. For example, NIAID has recently shut down a series of model organism databases (VeuPathDB) within a few months. We are unaware of any system from NIH or other funders providing sunsetting guidelines, a home for defunct databases, or funding programs for data storage or minimal IT support of defunct resources. The ENCODE portal is one of the few that secured minimal funding after the end of the project, through a special grant program. To prepare for its own demise, a project can submit data to general data archives so at least the files are still available, and URLs or DOIs could be forwarded to such an archive. This is easier if the data portal relies from the start on standardized static files rather than special database servers and APIs. The portal can try transferring the data to another project, to an “open data” free cloud storage bucket. It can also find alternative funding sources e.g. from users of the data, commercial sponsors or voluntary contributions, which is the route taken by Flybase and Arabidopsis.org. Some databases have also switched from academic grant funding to a purely commercial licensing model, such as HGMD or COSMIC, which goes against the FAIR principles, but at least helps the data and resources to continue to exist. Overall, long-term survival of project-specific data portals currently has no general solution or approach. For the FAIR principles, one would hope that funders transition data portals to a minimum funding level that ensures the survival of the data itself or move them to another portal.

### Data portals outside of genomics

The need to provide FAIR access and integrate data from a wide range of sources is becoming true for many scientific disciplines in the twenty-first century, from geography to the social sciences^68^. For example, CDC WONDER (https://wonder.cdc.gov/) hosts a suite of epidemiological data, the NOAA National Centers for Environmental Information (https://www.ncei.noaa.gov/) provide access to a variety of climatological and environmental data, Simons Collaborative Marine Atlas Project (https://simonscmap.com/) and Copernicus (https://marine.copernicus.eu/) host and visualize oceanographic data, and the Inter-university Consortium for Political and Social Research (https://www.icpsr.umich.edu/web/pages/) collects, collates, and visualizes social and political science data. Ecology, much like genomics, is increasingly dependent on integrating larger and larger multimodal datasets, moving towards “ecoinformatics”^69^. In geography, the term “spatial cyberinfrastructure” has been coined to refer to the integration of traditional experimental work with dispersed environmental sensors^70^. These domains face the same challenges that we have addressed in this article: metadata schema and flexibility, access and re-use, and the software, storage, and personnel costs of a data portal^69^. Therefore, while focused on genomics data portals, our lessons should be generally applicable across scientific disciplines.

## Conclusion

Data portals are important in making the data in data repositories and DCCs accessible. But designing and implementing a data portal that makes its data findable, easily accessible, interoperable, and reusable (or FAIR) is a difficult task, even for the expansive National Institutes of Health Common Fund Data Ecosystem^71^. These difficulties arise from a multitude of sources: research data is constantly changing; research labs are busy and data archival is not a big priority for them; turnover of graduate students and postdocs is high; and funders shy at the costs. Even industry IT projects with better defined and more stable use cases are known to overrun deadlines and budgets.

This article has sought to list out the issues that data portals face may face in during their early development and planning and to suggest some general guidelines to address these issues:

- We have found that data portals are easier to run when funders have set clear rules for data submission and adequately enforce and explain them to submitting labs.
- A list of the problems that the data portal is supposed to address and edge cases that should not be solved, should drive the metadata schema and the user interface.
- Data portals need a set of data wranglers armed with a strong but flexible metadata schema to collect the information necessary to make data reusable. Data wranglers are also crucial for helping guide data generators through the submission process.
- A data portal should employ UX designers and engineers who should work together to iteratively create data systems and interfaces to make the data findable and accessible in an intuitive manner. These developments should happen in small steps based on user demand and interest.
- Data portals should use existing or define new standard file formats and metadata fields which makes data interoperable with existing tools and workflows. Such interoperability combined with stable file formats can help the data long outlive its portal.
- Ideally, data portals can reuse existing consent rules, software, use cases, user interfaces and data curation standards from other, similar projects.

While we have focused on genomics and biomedical data, we believe that these lessons are applicable to nearly all scientific disciplines. Data portals are a cornerstone of data sharing and improving their design from the start should help make the data more findable, accessible, interoperable, and reproducible. Designing data portals from the start with these lessons in mind will also make the code, tooling, and procedures underlying the portals themselves more shareable between projects.

## Supplementary information

Supplemental information
