# Supplementary material for: Making genomic data FAIR through effective Data Portals
Source: Sci Data. 2025 Nov 28;12:1872. doi: 10.1038/s41597-025-06142-x (PMC12663326; doi:10.1038/s41597-025-06142-x)
Supplement: Supplementary file 1 — Supplemental information [file 41597_2025_6142_MOESM1_ESM.pdf]

Supplemental Information

Current Landscape

In this supplemental section, we provide an overview of the current landscape of data repositories and data coordination centers. The current landscape is represented by several geographically distinct organizations that fund databases of all three kinds, and an increasing number of focused DCCs and Data Portals created to meet the demand for niche technological, clinical, and scientific subsets. As earlier defined, data repositories accession and make available a wide range of data submitted to them. DCCs perform an equally important role but are more focused on a specific project or funding initiative. Data Portals do not always provide accessions but, similar to DCCs, bring together a focused subset of data and metadata. Several of the major organizations that maintain data repositories and DCCs are the National Institutes of Health (NIH) in the United States, the European Molecular Biological Laboratory - European Bioinformatics Institute (EMBL-EBI) based in the United Kingdom, and the DDBJ (DNA DataBank of Japan), based in Japan.

Data Repositories

NCBI - NLM - NIH

GenBank is a long-standing NIH-funded database for storing sequences such as assembled genome sequences, mRNAs, and ESTs. As of June 2022, GenBank contained over 1.8 billion distinct WGS sequences.

The Gene Expression Omnibus (GEO) is funded by the NIH and focuses on functional genomics data. GEO originally started as a database for storing microarray gene expression data, but has evolved to cover first bulk and now single-cell transcriptomics data as well. As of July 2022, GEO currently holds data and metadata on over 5 million separate samples. GEO employs curators to help submitters fill out metadata and upload files and will collect and curate data that can be analyzed together into GEO DataSets. The GEO metadata standard is quite limited, however, which means that even with the help of curators there is often limited information available for each GEO Series. Additionally, even though GEO curates some of these “Series” submissions into DataSets many users come to GEO through the Series accessions and will not see these curated DataSets. The files available for a Series are extremely variable, with some labs providing a full suite of analysis files while others may offer an expression matrix.

EMBL-EBI

The European Nucleotide Archive (ENA) is the European counterpart to GenBank, with a broader scope of data and metadata. It stores not only annotated sequence data but also the

associated project and sample metadata. As of July 2022, ENA contained 2.7 billion distinct sequences.

ArrayExpress is a large, open-submission data repository accepting high-throughput functional genomics data funded by EMBL-EBI. It is hosted at the EBI data centers in the UK or through the EMBL-EBI Cloud Portal. At the time of writing, ArrayExpress is being merged into the BioStudies database and will be retired by the end of 2022. As of July 2022, ArrayExpress currently has data and metadata on over 2.6 million distinct assays. ArrayExpress carries out similar curation to GEO, where curators work with submitters to improve the metadata of submissions, although ArrayExpress differs in that the requirements about files, formats, and metadata required for submission are more clear.

DDBJ Center

The DDBJ Sequence Read Archive (DRA) performs a similar function to both ENA and GenBank, being a data repository that broadly stores NGS data and metadata. It operates from Mishima, Japan, and functions to issue internationally recognized unique accession numbers and increase the accessibility and findability of data. As of July 2022, the DRA held data and metadata on over 2.7 billion distinct sequences.

Collaboration in Data Repositories - INSDC

NCBI, EMBL-EBI, and the DDBJ Center collaborate heavily with each other and other organizations to capture, exchange, and share data records. One archetypical example of their collaboration is the International Nucleotide Sequence Database Collaboration (INSDC). The INSDC is a long-standing initiative between the National Center for Biotechnology (NCBI), a division of the National Library of Medicine at the NIH, EMBL-EBI, and the DDBJ Center. It established data standards, formats, and procedures for data and metadata, and covered a wide range of data including NGS, capillary reads, annotated sequences, and contextual sample and study metadata. Importantly, it also ensured that any data entered into one of the organization’s databases is openly available, findable, and accessible in all formally linked databases. This includes GenBank, ENA, and the DRA, but not GEO or ArrayExpress. Data is exchanged between the organizations daily, which results in virtually the same sets of data. This is a representative example of how organizations can work together to ensure that data is findable, accessible, interoperable, and reusable (FAIR). As the number of data repositories, DCCs, and Data Portals increases, collaborations between organizations and teams is essential for ensuring FAIR principles are upheld in the data landscape.

Data Coordination Centres and Data Portals

NCBI - NLM - NIH

dbGaP is an NIH-funded DCC that focuses on data that requires restricted access, such as personally identifiable patient data. The accessioned data and metadata is not openly available

but may be made available on request to the centralized NIH Data Access Committee (DAC). As of July 2022, dbGaP held 356,522 expression assays.

The ENCODE Project (https://www.encodeproject.org/), focused on assaying and cataloging functional DNA elements outside of gene encoding regions, was started in 2003 with a pilot project studying just a few regions of the human genome. ENCODE has recently entered its fourth phase, and now covers the entire human and mouse genomes with over 50 different assays. While the ENCODE project is broad in scope in terms of goals and data types, it restricts data submissions to labs funded through the same initiative. ENCODE data is housed on Amazon Web Services (AWS). Data deposited in ENCODE is made available immediately, with limited options for managed-access data or pre-publication privacy. Earlier iterations offered grace periods for the data-generating lab to publish first, but these stipulations are gone in the latest version of ENCODE.

EMBL-EBI

The European Genome-Phenome Archive (EGA) is an EMBL-EBI-funded DCC focusing on data that requires restricted access, analogous to dbGaP funded by the NIH. Unlike dbGaP, EGA requires submitters to name a DAC for their data since the EGA does not act as a centralized DAC. In this way, EGA functions as a platform connecting individual DACs to users who request access to the data. EGA covers a broad range of data types, including but not limited to genetic, phenotypic, and clinical data. As of July 2022, the EGA hosted 8,366 datasets and 5,490 studies.

Single Cell Expression Atlas (SCEA) is an EMBL-EBI-funded DCC focusing on multi-species high-throughput single-cell expression data and visualization. SCEA sets standards of metadata and offers useful tooling such as interactive organ anatomograms, standardized AnnData objects, and *in situ* visualization of expression matrices. In addition, they work heavily with the single-cell community to develop appropriate metadata standards and useful UI changes. As of March 2022, SCEA contained 304 distinct single-cell RNA-Seq studies.

Other Organizations

The Human Cell Atlas (HCA) Data Coordination Platform (DCP, https://data.humancellatlas.org/) is funded through the Chan-Zuckerberg Initiative (CZI) and hosted on Google's Cloud Platform. It is an open-access data portal that takes a heavily-curated approach to data submission for single-cell and bulk RNA sequencing data. The DCP curates its data in two ways: (1) eligibility criteria focusing on datasets conducive to building a reference atlas, and (2) a detailed metadata standard that describes the entities and processes used to generate that data. While the standard is quite comprehensive, it has the tradeoff of typically requiring manual intervention on the part of data curators to assist submitters in properly completing it. With a heavy emphasis on FAIRification, this brings together single-cell sequencing data in an interoperable and reusable manner, allowing users to easily find and integrate their data with existing open data. The HCA DCP hosted 267 datasets as of July 2022.

Supplementary Tables

Author	Data Repositories, DCCs, and Data Portals worked on
Matthew L Speir	UCSC Stem Cell Hub, Human Cell Atlas Data Coordination Platform (HCA DCP), UCSC Cell Browser
Wei Kheng Teh	HCA DCP
Tim Harris	Dockstore, AnVIL
Marc Perry	modENCODE, International Cancer Genome Consortium (ICGC), UCSC Cell Browser
Parisa Nejad	UCSC Stem Cell Hub, HCA DCP
Rachel Schwartz	HCA DCP, UCSC Cell Browser
Clay Fischer	UCSC Stem Cell Hub, HCA DCP
Brian T Lee	ENCODE
Benedict Paten	HCA DCP, Human Pangenome Reference Consortium, BioData Catalyst, AnVIL, Dockstore
W James Kent	ENCODE, modENCODE, UCSC Stem Cell Hub, HCA DCP
Maximilian Haeussler	UCSC Stem Cell Hub, HCA DCP, UCSC Cell Browser

Table S1: A list of the authors and the DCCs they have contributed to in the past.

Acronym	Full name	Accepted data	Type	Controlled or Open	URL
4DN	4D Nucleome	Nucleomics sequencing and microscopy data	DCC	Open	https://data.4dnucleome.org/
ArrayExpress	ArrayExpress	High-throughput functional genomics	Data Repository	Open	https://www.ebi.ac.uk/arrayexpress/
BIL	Brain Image Library	Microscopy data focusing on the brain	DCC	Open & Controlled	http://www.brainimagelibrary.org/

BioData Catalyst	BioData Catalyst	Managed access data focusing on NHLBI (Heart, Lung, Blood Institute) datasets	DCC	Controlled	https://biodatacatalyst.nhlbi.nih.gov/
CSER	Clinical Sequencing Evidence-Generating Research	Genomic & Clinical Data	Coordination Center	Controlled	https://cser-consortium.org/
DANDI	Distributed Archives for Neurophysiology Data Integration	Neurophysiology data including electrophysiology, optophysiology, and behavioral time-series.	Data Portal	Open	https://www.dandiarchive.org/
dbGaP	Database of Genotypes and Phenotypes	Controlled genotype-phenotype data (GWAS, phenotype, SRA)	DCC	Controlled	https://www.ncbi.nlm.nih.gov/gap/
DDBJ	DNA DataBank of Japan	Annotated sequences	Data Repository	Open	https://www.ddbj.nig.ac.jp/index-e.html
EGA	European Genome-phenome Archive	Array-based, sequencing and phenotype data	DCC?	Controlled	https://ega-archive.org/
ENA	European Nucleotide Archive	Annotated sequences with associated project level and sample level metadata	Data Repository	Open	https://www.ebi.ac.uk/ena/browser/home
ENCODE	Encyclopedia of DNA Elements	Raw and ground-level analysis data	DCC	Open	https://www.encodeproject.org/

	GenBank	Annotated sequences	Data Repository	Open	https://www.ncbi.nlm.nih.gov/genbank/
GEO	Gene Expression Omnibus	High-throughput gene expression data and hybridization arrays, chips, microarrays	Data Repository	Open	https://www.ncbi.nlm.nih.gov/geo/
HCA DCP	Human Cell Atlas Data Coordination Platform	Single-Cell/Single-Nucleus RNA Sequencing	DCC	Open	https://data.humancellatlas.org/
HPRC	Human Pangenome Reference Consortium	Sequencing data from members of the HPRC	DCC	Open	https://humanpangenome.org/
HuBMAP	Human BioMolecular Atlas Program	High-throughput imaging and omics assays	DCC	Open	https://commonfund.nih.gov/HuBMAP
ICGC (defunct)	International Cancer Genome Consortium	Cancer Genomics	Data Repository	Open & Controlled	https://dcc.icgc.org/
NACC	National Alzheimer's Coordinating Center	Alzheimer's Disease Data	Coordination Center & Repository	Controlled	https://naccdata.org/
NeMO	Neuroscience Multi-omic Archive	Omics data generated from the BRAIN Initiative and related brain research projects	DCC	Open & Controlled	https://nemoarchive.org/
SCEA	Single Cell Expression Atlas	Curated single-cell datasets with visualizations and standardized files	DCC	Open	https://www.ebi.ac.uk/gxa/sc/home

UCSC SC Hub	UCSC Stem Cell Hub	Stem cell data for labs funded through the CIRM Center for Excellence in Stem Cell Genomics	DCC	Open & Controlled	https://cirm.ucsc.edu/
	COVID-19 Cell Atlas	Expression matrices and visualizations of COVID-19 and matched healthy donors	Data Portal	Open & Controlled	https://www.covid19cellatlas.org/

Table S2: A list highlighting some current genomics data repositories, DCCs, and data portals along with the data they accept, what type of resource they are, and the accessibility of the data they house.

Supplemental Figures

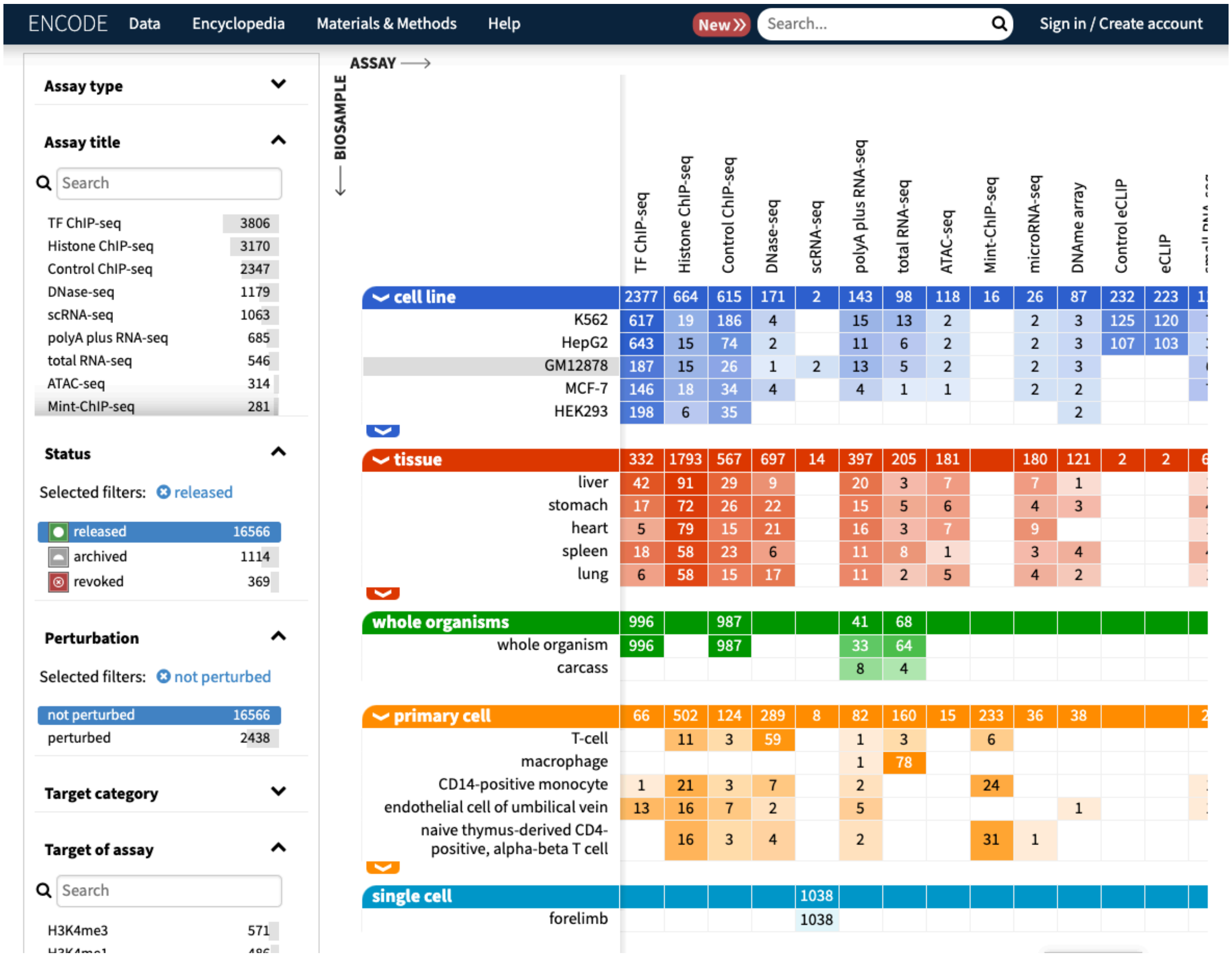

Figure S1: ENCODE Project matrix, *https://www.encodeproject.org/matrix/?type=Experiment. ENCODE provides an experiment matrix view of their data. The matrix at the center of the screen can be filtered using the facets along the left side. As filters are applied, the matrix will shrink to display only those matching results. Clicking a cell in the matrix will display a list of matching files.*

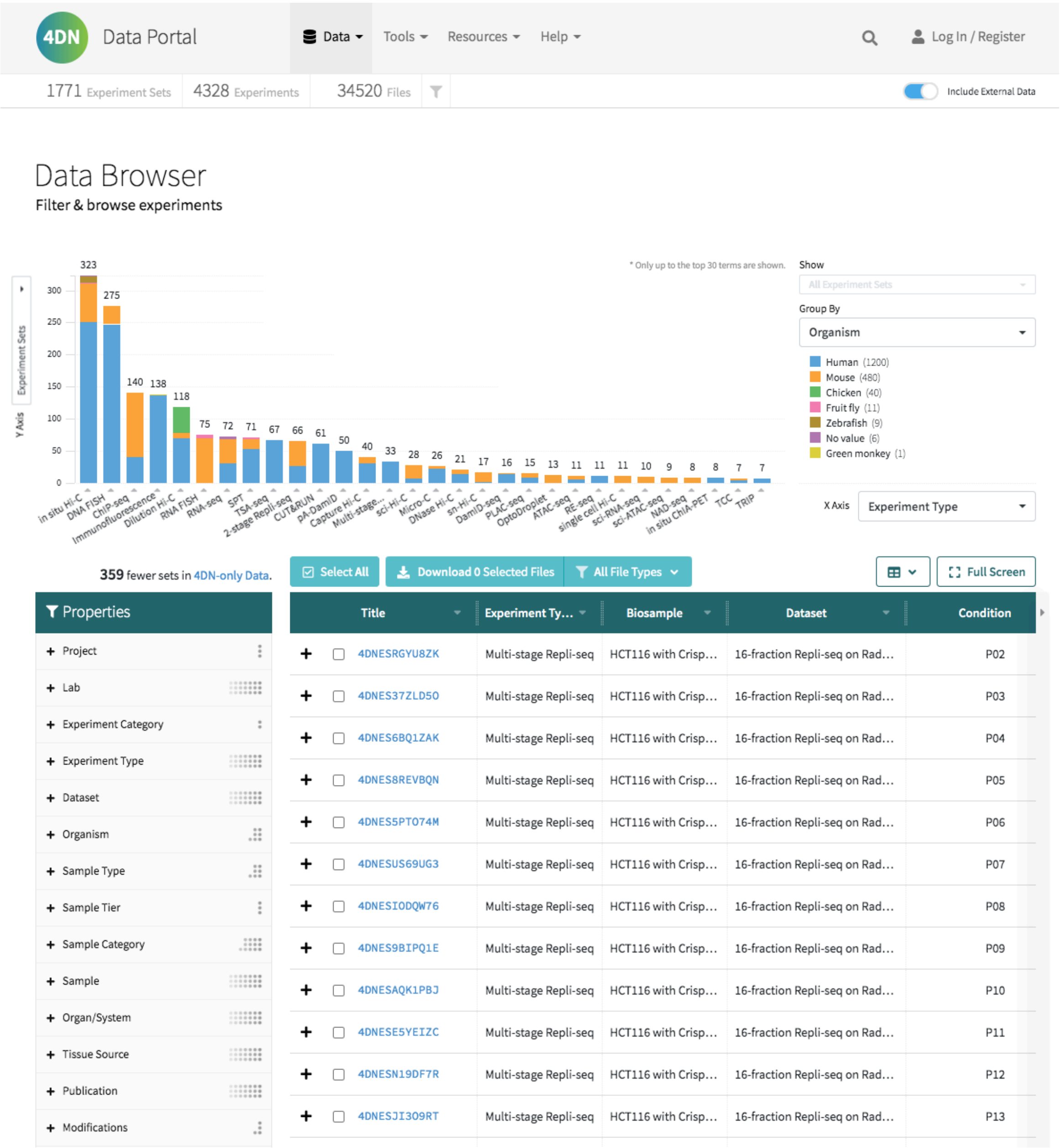

Figure S2: 4D Nucleome Data Browser, *https://data.4dnucleome.org/browse. A bar graph at the top shows the species breakdown for each assay. Below that is a list of files, which can be filtered using numerous metadata properties. Files can be selected and downloaded using checkboxes. Clicking on a file name will provide more details about the provenance of the file.*

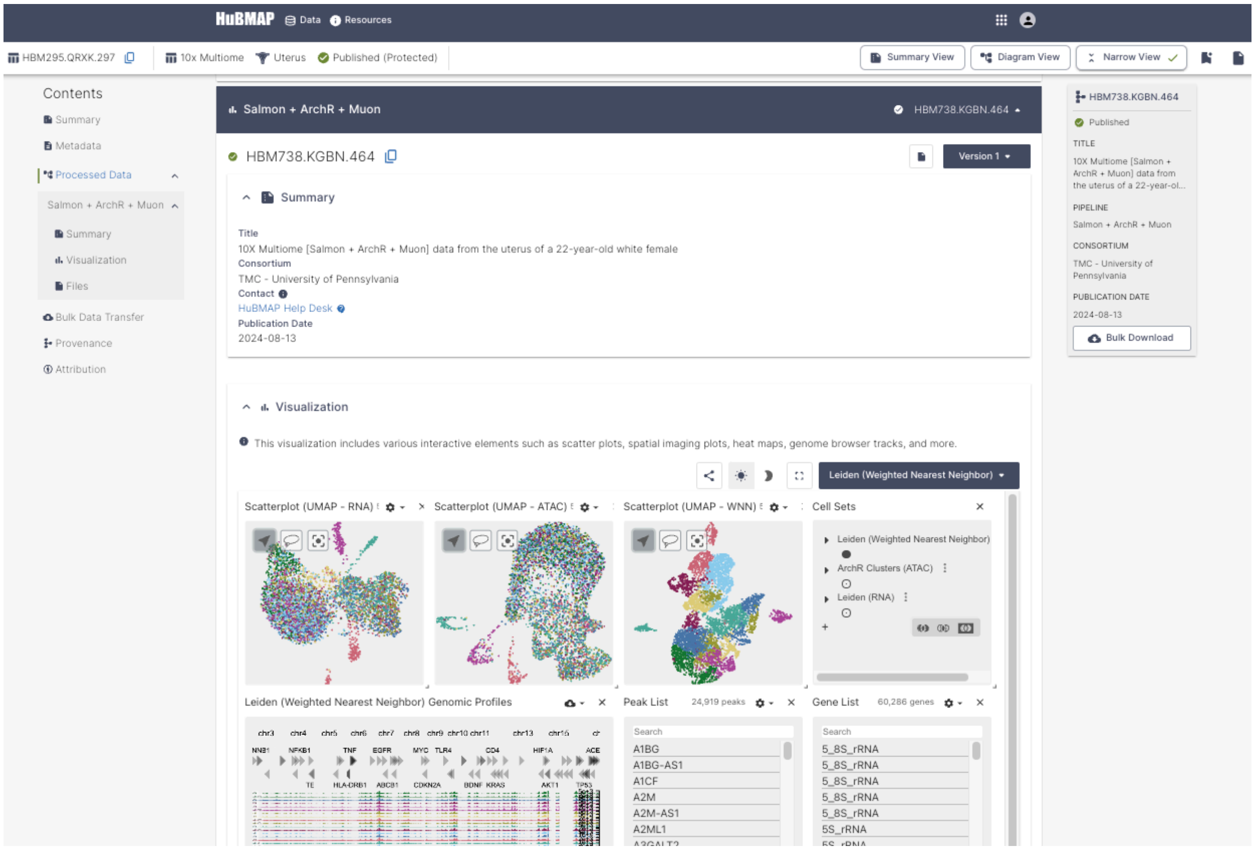

Figure S3: HuBMAP Dataset Details Page. *Data in HuBMAP can be browsed by dataset. A dataset description page includes several details about that dataset, including links between files, metadata, and visualizations of the processed data.*

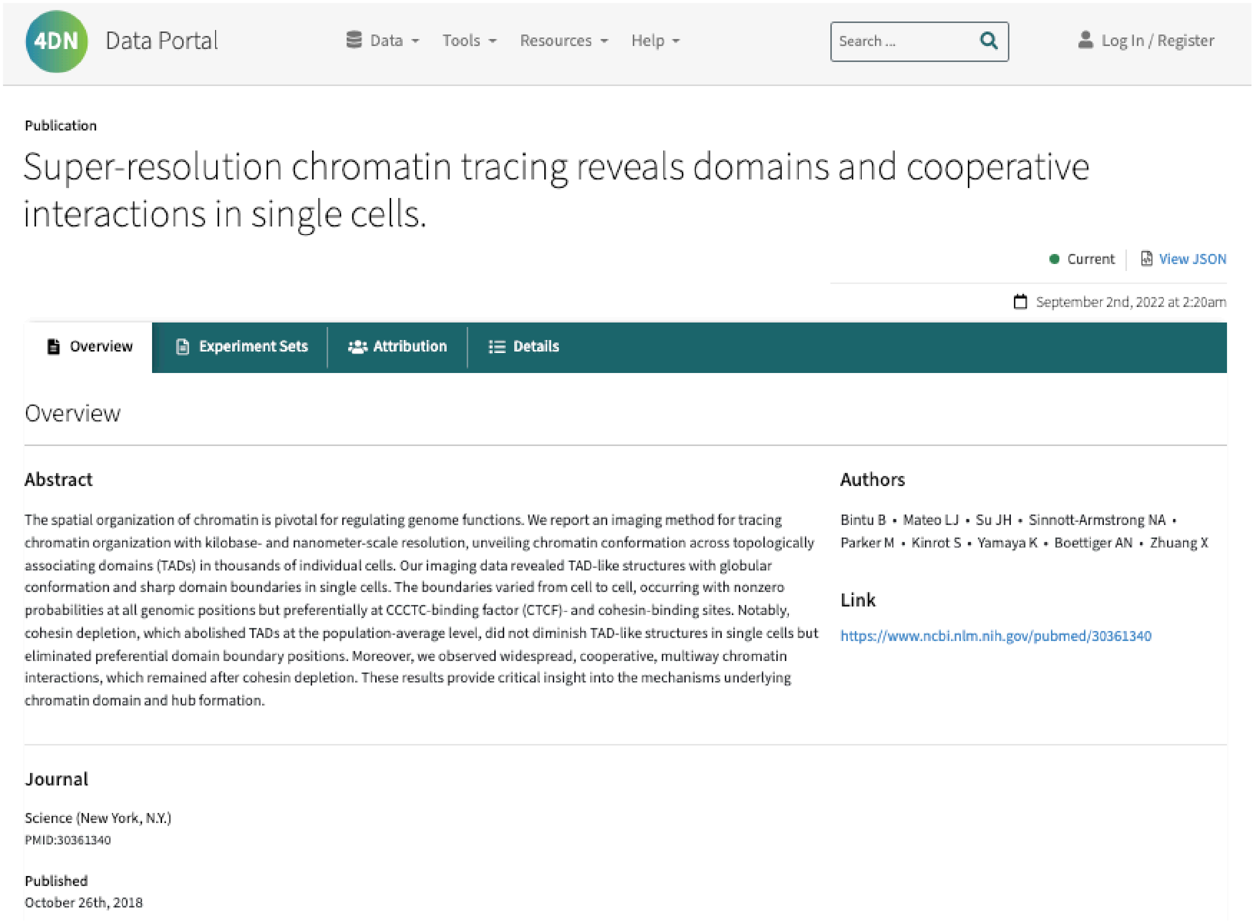

Figure S4: 4D Nucleome Dataset Details Page. *Data can be browsed by publication rather than by file or assay type. Clicking on a publication in this mode first brings users to the publication abstract. Different tabs below the publication title contain details about the submitters and accessions of any data linked to this publication.*

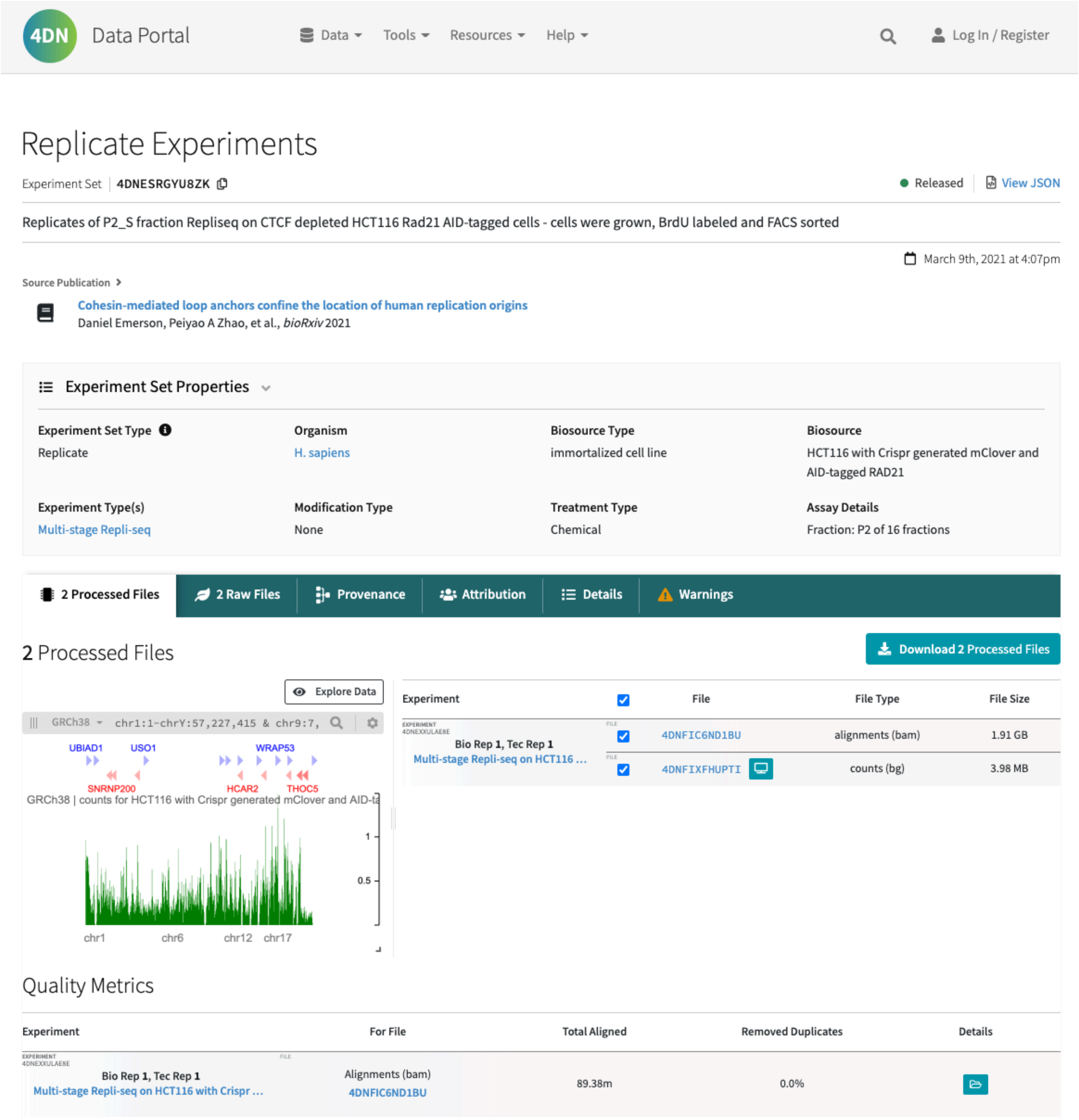

Figure S5: 4D Nucleome File Details Page. *Clicking on a file in the 4DN Data Browser (**Figure S2**) will bring up a page of file metadata. Different tabs contain information about process files derived from the original data, file provenance outlining analysis processes applied to the original files, and any quality warnings about the files.*

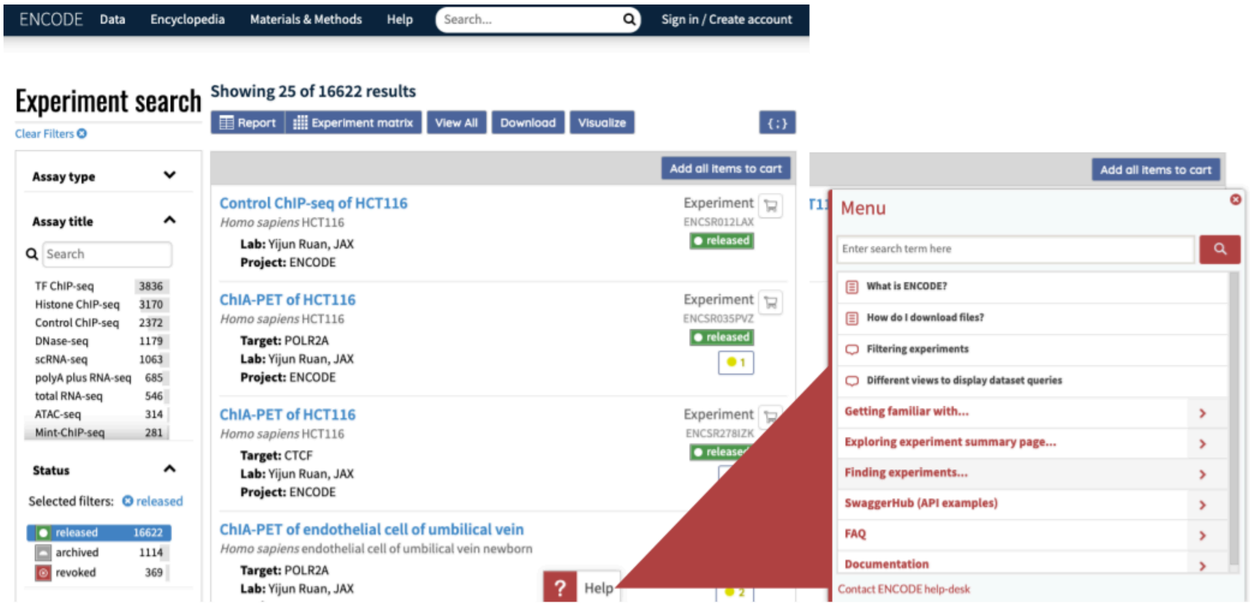

Figure S6: ENCODE Help Pop-up. *On all pages of the ENCODE website, there is a small "Help" button at the bottom of the screen. Clicking this button will bring up a list of common queries, links to various help pages, and the option to search all of their help pages. If none of those answer a user's question, at the bottom of the pop-up, there is a way to contact the ENCODE help desk.*
